# Probing the Importance of the G-Quadruplex Grooves for the Activity of the Anti-HIV-Integrase Aptamer T30923

**DOI:** 10.3390/ijms21165637

**Published:** 2020-08-06

**Authors:** Veronica Esposito, Francesca Esposito, Antonietta Pepe, Isabel Gomez Monterrey, Enzo Tramontano, Luciano Mayol, Antonella Virgilio, Aldo Galeone

**Affiliations:** 1 Department of Pharmacy, University of Naples Federico II, 80131 Napoli, Italy; verespos@unina.it (V.E.); isabelmaria.gomezmonterrey@unina.it (I.G.M.); mayoll@unina.it (L.M.); 2 Department of Life and Environmental Sciences, University of Cagliari, Cittadella Universitaria SS554, 09045 Monserrato (CA), Italy; francescaesposito@unica.it (F.E.); tramon@unica.it (E.T.); 3 Department of Science, University of Basilicata, 85100 Potenza, Italy; antonietta.pepe@unibas.it

**Keywords:** G-quadruplex, 8-methyl-2′-deoxyguanosine, aptamers, integrase, HIV

## Abstract

In this paper, we report studies concerning four variants of the G-quadruplex forming anti-HIV-integrase aptamer T30923, in which specific 2′-deoxyguanosines have been singly replaced by 8-methyl-2′-deoxyguanosine residues, with the aim to exploit the methyl group positioned in the G-quadruplex grooves as a steric probe to investigate the interaction aptamer/target. Although, the various modified aptamers differ in the localization of the methyl group, NMR, circular dichroism (CD), electrophoretic and molecular modeling data suggest that all of them preserve the ability to fold in a stable dimeric parallel G-quadruplex complex resembling that of their natural counterpart T30923. However, the biological data have shown that the T30923 variants are characterized by different efficiencies in inhibiting the HIV-integrase, thus suggesting the involvement of the G-quadruplex grooves in the aptamer/target interaction.

## 1. Introduction

Acquired immune deficiency syndrome (AIDS) is a disease of the human immune system, whose etiological agent is the human immunodeficiency virus (HIV), belonging to the *Retroviridae* family. Nowadays, about 35 million people are infected with HIV worldwide and considering 2 million new infections and about 1.6 million deaths from this disease per year, AIDS is still a global emergency. The development of highly efficient anti-retroviral drugs has allowed most infected people to live with HIV as a chronic disease [1]. However, emergent drug resistance highlights the need to develop further effective unconventional ligand molecules, for example, aptamers or monoclonal antibodies. In particular, aptamers are DNA or RNA ligands able to compete with antibodies in therapeutic, analytic and diagnostic applications, thanks to their capacity to interact with high affinity and specificity with a given target molecule [2]. These ligands can be discovered fortuitously, through several combinatorial approaches collectively called Systematic Evolution of Ligands by Exponential Enrichment (SELEX) or by other techniques [3]. Considering that the ability of these ligand molecules to adopt a specific and thermodynamically stable three-dimensional structure is one of the most important characteristics, it is not particularly surprising that several aptamers are characterized by G-rich sequences and fold in a G-quadruplex (G4) conformation [4]. In fact, thanks to their stacked G-tetrads (each stabilized by eight H-bonds) and a monovalent metal ion between them, this DNA or RNA conformation is considered among the most stable ones.

G4 aptamers have been developed against some crucial proteins involved in the HIV life cycle. For example, by using an approach called synthetic unrandomization of randomized fragments (SURF), the G4 aptamer ISIS 5320 (phosphorothioate T_2_G_4_T_2_) was discovered [5]. This aptamer, which adopts a parallel tetramolecular G4 structure interacting with the HIV glycoprotein gp120, has shown a sub-micromolar inhibition of HIV-1. Further aptamers targeting the HIV-1 entry through gp120 interaction are formed by Hotoda’s hexamer sequence TG_3_AG (and derivatives bearing lipophilic groups at the 5′-ends) [6] adopting a parallel tetramolecular G4 structure containing an A-tetrad [7]. However, Hotoda’s aptamer derivatives are also known to form parallel bimolecular [8] or monomolecular G4 structures [9] based on suitable bifunctional or tetrafunctional linkers [10]. In order to isolate DNA aptamers against the HIV-reverse transcriptase, SELEX approaches have been exploited [11]. These selections have led to the G4 aptamer 93del, which folds in an unusual dimeric interlocked G4 structure [12]. The HIV-1 integrase is a further target protein for which G4 aptamers have been developed. These include a group of G- and T-containing oligonucleotides forming structures strictly related to each other, namely T30923 [(G_3_T)_4_] and T30175 [(GTG_2_T(G_3_T)_3_], and their partially phosphorothioate versions, T30695 and T30177, respectively [13,14,15,16,17,18,19,20]. These sequences have been proven to fold in a head-to-head dimer of two identical 5′-5′ end-stacked parallel G4 structures, each characterized by three G-tetrads and three or four loops consisting of only one thymidine [21,22,23,24]. Importantly, this group of G4 aptamers includes the first HIV-1 integrase inhibitor tested in clinical trials, namely T30177 (Zintevir, developed by Aronex Pharmaceuticals in 1996) [25].

**Figure 1:**
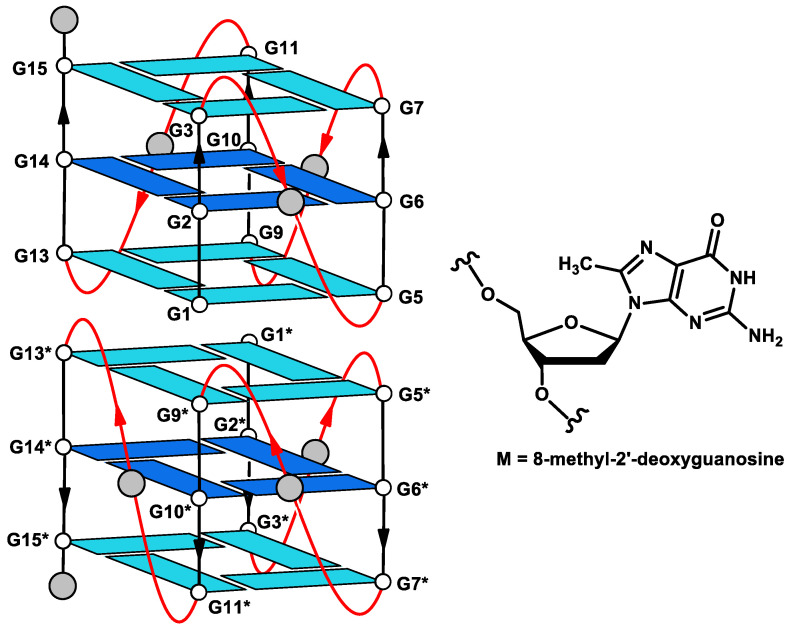
Schematic representation of the parallel-stranded G4 dimer structure of Table 1. (INT-nat). Residues in dark blue have been singly replaced by M residues in the T30923 variants (Table 1). Thymidines are indicated by grey circles.

Undoubtedly, the development of more efficient new derivatives has effectively benefited from investigations on the structural aspects of the aptamer/target protein interaction, particularly if the loops of the G4 aptamer were involved. For example, the finding that the small loops of the “chair-like” G4 thrombin binding aptamer (TBA) are definitely involved in the interaction with the target protein allowed the development of derivatives with improved anticoagulant activities [26,27]. Furthermore, in two studies aimed at investigating the interaction of T30923 with the integrase and improving the biological properties of T30175, we have recently highlighted the importance of the loops for the aptamer/target interaction and for the integrase-inhibiting activity [28,29]. Unusually, although the relevance of the interaction between small molecules and the grooves of biologically significant G4 structures for the development of groove-binding drugs is well known [30], investigations focused on the role of the grooves in the aptamer/target protein interaction are still relatively scant. By exploiting an approach similar to that successfully used to validate the groove binding of small-molecule drugs with G4 structures [31,32], in this paper, we describe a strategy to evaluate the involvement of the G4 grooves for the integrase-inhibiting activity of the aptamer T30923. For this purpose, by using the single residue replacement approach of some 2′-deoxyguanosines in specific positions with 8-methyl-2′-deoxyguanosine residues (M), we have prepared four T30923 variants with the aim to exploit the methyl group as a “steric probe” (Table 1, Figure 1).

## 2. Results

### 2.1. NMR Spectroscopy

**Figure 2:**
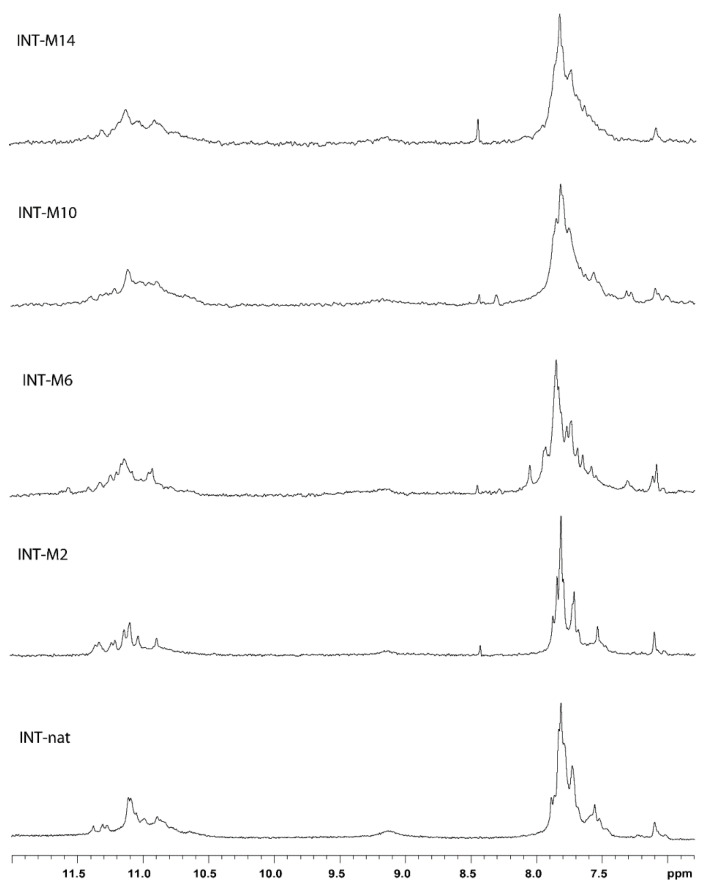
Imino and aromatic proton regions of the ^1^H-NMR spectra (500 MHz) of the T30923 analogues investigated. See Materials and Methods for experimental details.

A crucial point of this investigation is the preliminary confirmation that the modified oligodeoxynucleotides (ODNs) preserve the ability to fold in the parallel dimer G4 structure characteristic of the natural sequence (T30923) (Figure 1), in order to avoid any misinterpretation of the data concerning the biological activity, due to the presence of alternative conformations differing from the parent one. In order to address this point, the ODN analogues were analyzed by ^1^H-NMR and compared with their unmodified counterpart (Figure 2). A straightforward comparison of the imino proton regions (10.5–12.0 ppm) diagnostic of the presence of G4 structures indicates that, although slight expected differences between ^1^H-NMR spectra can be noted, due to the effect of the methyl group in different positions, the single modification changes the shape and the position of the imino proton resonances of the G-tetrads only negligibly, thus suggesting that the conformation adopted by the modified ODNs containing an M residue is similar to that of their natural counterpart. In particular, the fact that all ^1^H-NMR spectra show similar imino and aromatic chemical shift ranges and comparable relative peak intensities suggests that the new derivatives maintain the T30923 ability to fold in 5′-5′ end-to-end stacked dimers of parallel G4 structures.

### 2.2. CD Spectroscopy

**Figure 3:**
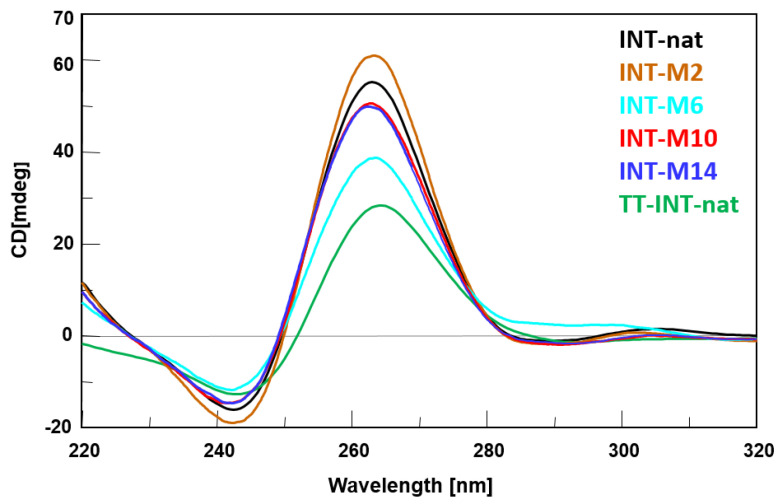
CD spectra of the T30923 analogues investigated. See Materials and Methods for experimental details.

With the aim of corroborating ^1^H-NMR data, the CD spectra of the modified T30923 aptamers were acquired and compared with their natural counterpart and with the ODN TT-(G_3_T)_4_ (TT-INT-nat) corresponding to the sequence of T30923, extended with two extra thymidines at the 5′-end, which have proven to prevent the formation of the 5′-5′ head-to-head dimer (Figure 3) [24]. Despite small differences in intensity, all profiles are almost superimposable on each other, showing a minor negative band at 242 nm and a major positive band at 263 nm, which are characteristic of parallel G4 structures in which all guanosines adopt *anti*-glycosidic conformations. CD data strongly suggest that modified sequences adopt G4 structures strictly resembling that of the parent aptamer T30923, in agreement with NMR results. Furthermore, the thermal stability of the modified G4 structures was determined by CD melting experiments. In order to test the effect of the modification on the G4 thermal stabilities, CD heating profiles were acquired in the NMR buffer conditions (data not shown). The unchanged CD signal up to about 80 °C and the absence of a sigmoidal melting profile for all the modified sequences suggest that the high structural stability of the parent G4 structure was mostly preserved. However, some differences in the structural stability of the modified G4 structures could be observed by recording CD melting temperatures (T_m_) at very low potassium ion concentrations (5 mM KCl) (Table 1, Figure S1). The CD data obtained in these conditions still pointed to a rather high stability for all structures, although the modified ones showed lower T_m_s compared to the parent G4 complex. With the aim of evaluating the structural stabilities of the modified aptamers during the HIV-1 IN catalytic reaction (see below) we recorded CD heating profiles of the preformed structure (see Material and methods) (Figure S2) in the same buffer used for this assay [29]. Moreover, in this case, the unchanged CD signal up to 70 °C for all the modified aptamers indicates that in the biological assay conditions (37 °C) all G4 structures were retained.

The whole of the data indicated that the single replacement of some 2′-deoxyguanosines in specific positions with M residues does not significantly affect the ability to adopt very stable conformations. Therefore, although in all the modified G4s, a G-residue adopting an *anti*-glycosidic conformation was replaced with an M one, usually more prone to adopt a *syn* conformation, all structures preserved a noteworthy structural thermal stability.

### 2.3. Polyacrylamide Gel Electrophoresis (PAGE)

**Figure 4:**
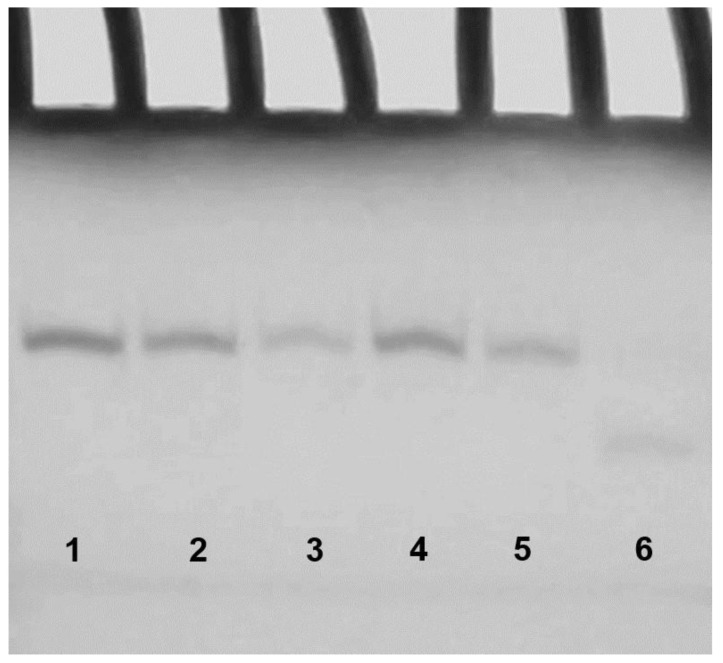
Polyacrylamide Gel Electrophoresis (PAGE) analysis of the T30923 analogues investigated. Lane 1: INT-M2; lane 2: INT-M6; lane 3: INT-M10; lane 4: INT-M14; lane 5: INT-nat; lane 6: TT-INT-nat. See Materials and Methods for experimental details.

It should be noted that CD experiments, even though rather informative, do not provide a clear evidence of the presence of dimers for the modified sequences. In order to address this point, we analyzed the modified sequences by Polyacrylamide Gel Electrophoresis (PAGE) (Figure 4) in comparison with the natural sequence INT-nat (which has already been proven to form a dimer) and TT-INT-nat (in which the dimer formation is prevented by the extra thymidines in 5′) [24]. The PAGE results clearly indicated that INT-nat and all ODNs containing an M residue show slower-migrating bands, indicative of dimeric structures, while TT-INT-nat shows a faster migrating band, thus pointing to the presence of a monomeric G4 structure. The PAGE results are in agreement with the data obtained by the other techniques, all pointing to the presence of G4 structures for the modified aptamers, strictly resembling that one adopted by the parent ODN.

### 2.4. Molecular Modeling (MM)

**Figure 5:**
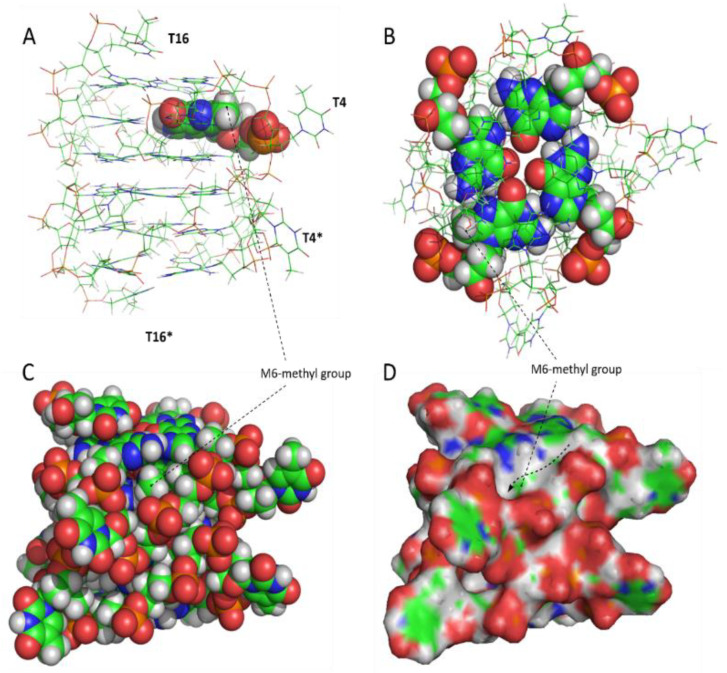
G-quadruplex structure adopted by INT-M6. (**A**) Side view of the stick model. The M-residue is reported in CPK. (**B**) Top view of the stick model. The central G-tetrad is in CPK. (**C**) Side view of the CPK model. (**D**) Side view of the surface model. The dashed line with an arrow indicates the groove in which the methyl group is positioned. Heavy atoms are shown with different colors (carbons, green; nitrogens, blue; oxygens, red; hydrogens, white; phosphorus, orange).

Molecular models for all the modified aptamers have been built (Figure 5 and Figures S3–S5). As expected, the replacement of a hydrogen atom with a methyl group does not significantly affect the general folding of the derived analogues, compared to the original T30923, as already suggested by NMR data, all exhibiting parallel G4 structures adopting a right-handed helical alignment. However, it is noteworthy that all the tetrads of the modified aptamers do not appear to be completely planar due to moderate buckles as a result of the steric hindrance of the methyl group in position 8 of the central guanines, as suggested by the slight signal broadening of the ^1^H-NMR spectra of most of the derivatives.

### 2.5. Effect of T30923 Derivatives on the HIV-1 IN LEDGF-Independent Activity

It has been reported that HIV-1 IN activities can be inhibited by compounds that act on the active site [33,34,35] or allosterically. In fact, many small molecules [36], peptides [37], natural compounds [38,39,40] and G4 aptamers [41] are known to act as inhibitors. With the aim of evaluating the involvement of the grooves of the G4 structure in the aptamer/target protein interaction and, in turn, in the HIV-1 IN activity, the M-containing oligonucleotides were tested on the HIV-1 IN catalytic activities, using the approved IN strand transfer inhibitor Raltegravir as a control. The results showed that all the modified analogues potently inhibited the HIV-1 IN activities (Table 1), also in accordance with the data present in the literature. In particular, INT-M2, INT-M10 and INT-M14 inhibited the HIV1-IN LEDGF-independent activities with IC_50_ values ranging from 0.228 to 0.280 μM, thus showing lower potencies than their unmodified counterpart. On the other hand, INT-M6 was the most active oligonucleotide, showing an IC_50_ value of 0.096 μM, similar to that one reported for INT-nat.

## 3. Discussion

In this study, the structural and biological properties of analogues of aptamer T30923 containing 8-methyl-2′-deoxyguanosine residues were investigated. The design of these analogues was based on previous studies indicating that tetramolecular parallel G4 complexes were able to tolerate the presence of an 8-methyl-2′-deoxyguanosine residue in specific positions, thus arranging the methyl groups in the medium-size grooves, without significant structural changes [42,43,44]. The collected NMR, CD, PAGE and Molecular Modeling (MM) data strongly suggest that all T30923 analogues adopt G4 structures strictly resembling that of the unmodified aptamer. However, the results concerning the inhibition of the HIV-1 IN catalytic activity indicate that the M-containing T30923 analogues show different efficiencies. In fact, while INT-M6 retains an inhibiting activity quite similar to that of the original aptamer, INT-M2, INT-M10 and INT-M14 show activities lower than that of T30923. In an investigation concerning analogues of the anti-HIV-IN aptamer T30695 (the version of T30923 with two phosphorotioate linkages) containing 5-propinyl-dU or 5-amino-dU, some authors in 2000 observed a relationships between the thermal stability and the inhibiting activity [18]. A simple comparison between the melting temperatures shown by these T30695 derivatives and the CD heating data collected in the case of the M-containing T30923 analogues indicates that our modified aptamers prove to be more stable, particularly taking into account the low buffer K^+^ concentrations necessary to obtain reliable measurements of their melting temperatures (Table 1). Most importantly, concerning the M-containing T30923 analogues, no clear stability–activity relationships emerged from the collected data, thus indicating that, in our case, the lower activities observed could not be ascribed to the minor thermal stabilities shown by the modified aptamers, compared to their natural counterpart INT-nat. Indeed, the less stable aptamer INT-M6 shows the highest activity, comparable to that of the original aptamer, while INT-M2, INT-M10 and INT-M14 show IC_50_ values about three times higher than T30923. These results indicate that the steric hindrance of the methyl groups in positions 2, 10 and 14 negatively affects the HIV-IN inhibiting activity of the modified aptamers, thus suggesting the importance of some of the G4 grooves for the aptamer/target protein interaction. It has been suggested that T30923 interacts with HIV-IN similarly to 93del (a further dimer G4 forming aptamer able to inhibit the HIV-IN enzymatic activity) [41]. Some studies concerning the model of HIV-IN in complex with 93del propose the positively charged cavity at the dimer-of-dimers interface in the structure of the HIV1-IN tetramer, embracing the dimeric aptamer, as a suitable site for 93del binding [12,41]. Contrary to 93del, the thrombin binding aptamer (TBA) forming a chair-like G4 structure has been proven to interact with its target mostly through its loops rather than through the core of the G4 structure [45]. This information was indirectly confirmed by data concerning the anticoagulant activity of TBA analogues, which contain M residues, replacing the four guanosines adopting *syn* glycosidic conformations, one at a time or all together [46]. In these cases, the methyl groups are positioned in the major grooves of the G4 structure. Interestingly, all these TBA analogues preserved a significant anticoagulant activity, thus excluding the direct involvement of the G4 major grooves in the interaction with the thrombin. These considerations support the exploitation of the methyl group in the M residues as a steric probe to provide an insight into the interaction between a G4-aptamer and its target.

In fact, information concerning the main features of the aptamer/target interaction are of crucial importance in designing suitably modified analogues with improved properties but, at the same time, avoiding the drop of the high affinity and specificity acquired following the selection processes usually utilized to discover aptamers. In the context of combinational antiretroviral therapy (ART), which was formulated to circumvent the ability of HIV to quickly develop resistance to single-drug treatments, the information resulting from our investigations could be useful in designing new derivatives of aptamer T30923 against viral strains resistant to the original molecule.

## 4. Materials and Methods

### 4.1. Oligonucleotide Synthesis and Purification

The modified oligonucleotides reported in Table 1 were synthesized on an ABI 394 DNA synthesizer using solid phase β-cyanoethyl phosphoramidite chemistry at the 10 µmol scale. The modified monomer was introduced in the sequences using commercially available 8-methyl-2′-deoxyguanosine (N-iBu)-3′-CE phosphoramidite (ChemGenes, Wilmington, MA, USA). The oligomers were detached from the support and deprotected by treatment with concentrated aqueous ammonia at 55 °C overnight. The combined filtrates and washings were concentrated under reduced pressure, redissolved in H_2_O, analyzed and purified by high-performance liquid chromatography on a Nucleogel SAX column (Macherey–Nagel, 1000-8/46, Düren, Germany), using buffer A: 20 mM KH_2_PO_4_/K_2_HPO_4_ aqueous solution (pH 7.0) containing 20% (v/v) CH_3_CN and buffer B: 1 M KCl, 20 mM KH_2_PO_4_/K_2_HPO_4_ aqueous solution (pH 7.0) containing 20% (v/v) CH_3_CN; a linear gradient from 0 to 100% B for 45 min and a flow rate of 1 mL/min were used. The fractions of the oligomers were collected and successively desalted by Sep-Pak cartridges (C-18). The isolated oligomers were proven to be >98% pure by NMR.

### 4.2. NMR Spectroscopy

NMR samples were prepared at a concentration of about 1 mM, in 0.6 mL (H_2_O/D_2_O 9:1 v/v), buffer solution having 10 mM KH_2_PO_4_/K_2_HPO_4_, 70 mM KCl and 0.2 mM EDTA (pH 7.0). All the samples were heated for 5–10 min at 90 °C and slowly cooled (10–12 h) to room temperature. The solutions were equilibrated for 24–48 h at 4 °C. The annealing process was assumed to be complete when the ^1^H NMR spectra were superimposable on changing time. NMR spectra were recorded with a Varian Unity INOVA 500 MHz spectrometer. The 1D proton spectra of the samples in H_2_O were recorded using pulsed field gradient DPFGSE for H_2_O suppression [47]. ^1^H-chemical shifts were referenced relative to external sodium 2,2-dimethyl-2-silapentane-5-sulfonate (DSS).

### 4.3. CD Spectroscopy

CD samples of oligonucleotides reported in Table 1 were prepared at an ODN concentration of 50 µM using a potassium phosphate buffer (1 mM KH_2_PO_4_/K_2_HPO_4_, 5 mM KCl, pH 7.0) and submitted to the annealing procedure (heating at 90 °C and slowly cooling at room temperature). CD spectra of all quadruplexes and CD melting curves were registered on a Jasco 715 CD spectrophotometer. For the CD spectra, the wavelength was varied from 220 to 320 nm at 100 nm min^−1^ scan rate, and the spectra recorded with a response of 4 s, at 1.0 nm bandwidth and normalized by subtraction of the background scan with the buffer. The temperature was kept constant at 20 °C with a thermoelectrically controlled cell holder (Jasco PTC-348). CD melting curves were registered as a function of temperature (range: 20 °C–90 °C) for all G4s, annealed as previously reported, at their maximum Cotton effect wavelengths. To test the G4 thermal stabilities in the same conditions of the biological assay, samples of modified ODNs were prepared at an ODN concentration of 2 mM using a potassium phosphate buffer (1 mM KH_2_PO_4_/K_2_HPO_4_, 5 mM KCl, pH 7.0) and submitted to the annealing procedure. The preformed structures thus obtained have been diluted at 50 µM ODN in the same reaction buffer used for the integrase assay and tested in CD melting experiments. CD melting curves were registered as a function of temperature (range: 20 °C–70 °C) for all G4s at their maximum Cotton effect wavelengths. The CD data were recorded in a 0.1 cm pathlength cuvette with a scan rate of 30 °C/h.

### 4.4. Gel Electrophoresis

All oligonucleotides were analyzed by non-denaturing PAGE. Samples in the CD buffer (1 mM KH_2_PO_4_/K_2_HPO_4_, 5 mM KCl, pH 7.0) were loaded on a 20% polyacrylamide gel containing Tris–Borate–EDTA (TBE) 2.5× and KCl 5 mM. The run buffer was TBE 1× containing 10 mM KCl. For all samples, a solution of glycerol/TBE 10× was added just before loading. Electrophoresis was performed at 8 V/cm at a temperature close to 10 °C. Bands were visualized by UV shadowing. The difference in the band intensities is due to the different amounts of loaded samples of the various derivatives.

### 4.5. Molecular Modeling

The main conformational features of G4s adopted by the modified ODNs were explored by means of a molecular modelling study, using the CVFF force field [48,49]. The initial coordinates for the starting model of INT-nat were taken from the NMR solution structure of the dimeric G4 T30923 (PDB 2LE6_T30695) [23]. In each monomer, the structure of the G4 containing an 8-methyl-2′-deoxyguanosine residue was built using the Biopolymer building tool of Discover by deleting a canonical 2′-deoxyguanosine (or 2′-deoxyinosine for INT-M2) at the appropriate position one at a time and replacing it with an 8-methyl-2′-deoxyguanosine to generate the modified derivatives. The 2′-deoxyinosine residue in position 2 was replaced with a canonical 2′-deoxyguanosine one for INT-M6, INT-M10 and INT-M14. In total, two modified residues are present in each dimeric aptamer. The calculations were performed using a distance-dependent macroscopic dielectric constant of 4r and an infinite cutoff for non-bonded interactions to partially compensate for the lack of solvent used [50]. Using the steepest descent followed by conjugate gradient methods, the conformational energy of the complexes was minimized until convergence to a Root Mean Square (RMS) gradient of 0.1 kcal/mol. All the calculations were performed on a PC running Linux ES 2.6.9 using the INSIGHT II program (Accelrys, San Diego, CA, USA). Illustrations of structures were generated with Pymol (The PyMol Molecular Graphics System; Version 1.8; Schrödinger, LLC: New York, NY, USA).

### 4.6. Expression and Purification of Recombinant HIV-1 IN

Recombinant full-length 6×His tagged HIV-1 IN protein was expressed and purified as described previously [51]. Briefly, the protein was expressed in *Escherichia coli* strain BL21 (DE3). The purification was done using a Ni Sepharose column with an imidazole gradient from 20 mM to 500 mM concentration in a 50 mM HEPES (pH 7.5) buffer containing 1 M NaCl, 7.5 mM CHAPS and 2 mM β-mercaptoethanol. The fractions containing the protein were followed by heparin column purification with a NaCl gradient from 0 to 1 M concentrations. Fractions were pooled and stored in 15% glycerol at −80 °C.

### 4.7. HTRF Integrase LEDGF-Independent Assay

The HIV-1 IN assay that allows the measurement of the inhibition of the 3′-processing and strand transfer IN reactions was performed as described in Virgilio et al. (2018) [29].

## 5. Conclusions

Investigations concerning the main structural features of an aptamer affecting the interaction with the target and/or the biological activity are of crucial importance for its future development as therapeutic or diagnostic agent. In the present study, we exploited the methyl group of an 8-methyl-2′-deoxyguanosine residue as a steric probe to investigate the importance of the grooves for the inhibiting activity of a G4 anti-HIV-integrase aptamer, namely T30923, adopting a parallel-stranded G4 dimer structure, in which only anti-2′-deoxyguanosines occur. A series of four T30923 analogues were prepared in which the canonical 2′-deoxyguanosine residues involved in the formation of the central G-tetrad were replaced, one at a time, with an 8-methyl-2′-deoxyguanosine one. The data collected from several techniques suggest that all the modified aptamers adopt G4 structures very similar to that of their parent aptamer. However, although all the T30923 derivatives preserve the ability to inhibit the HIV-1 IN catalytic activity, they showed quite different efficiencies, thus suggesting a role of the G4 grooves in the interaction aptamer/protein-target. The whole of the data collected in the present study and those from other investigations concerning TBA analogues containing an M residue validate the utilization of the methyl group in 8-position as a steric probe in investigations of the G4-aptamer/target protein interaction. The information obtained by this approach could be particularly useful in assisting molecular modeling docking studies by focusing on specific G4 regions involved in the aptamer/target protein interaction interface.

## Figures and Tables

**Table 1:** Sequence, melting temperature (T_m_) and effect of the modified oligodeoxynucleosides (ODNs) on the HIV-1 integrase activity.

Oligonucleotide	Sequence	T_m_ (°C) ± 1	^a^ IC_50_ IN LEDGF-Independent Activity (µM)
INT-M2	5′-GMGTGGGTGGGTGGGT-3′	75	0.275 ± 0.025
INT-M6	5′-GGGTGMGTGGGTGGGT-3′	67	0.096 ± 0.001
INT-M10	5′-GGGTGGGTGMGTGGGT-3′	71	0.228 ± 0.031
INT-M14	5′-GGGTGGGTGGGTGGMGT-3′	75	0.280 ± 0.060
INT-nat (T30923)	5′-GGGTGGGTGGGTGGGT-3′	88	0.088 ± 0.003
Raltegravir	N.A.	N.A.	0.058 ± 0.002

^a^ Compound concentration required to inhibit the HIV-1 IN catalytic activities by 50% in the absence of LEDGF/p75 cellular cofactor; M = 8-methyl-2′-deoxyguanosine; T_m_ = melting temperature in the 5 mM KCl buffer; Raltegravir has been used as a reference; N.A. = not applicable (see Materials and Methods for details).

## Supplementary Materials

The following are available online at https://www.mdpi.com/1422-0067/21/16/5637/s1. Figure S1: CD melting profiles in potassium buffer. Figure S2: CD heating profiles in the buffer of the integrase assay. Figure S3: Molecular models of INT-M2. Figure S4: Molecular models of INT-M10. Figure S5: Molecular models of INT-M14.
