# Supplementary material for: Probing the Importance of the G-Quadruplex Grooves for the Activity of the Anti-HIV-Integrase Aptamer T30923
Source: Int J Mol Sci. 2020 Aug 6;21(16):5637. doi: 10.3390/ijms21165637 (PMC7460552; doi:10.3390/ijms21165637)
Supplement: Supplementary file 1 [file ijms-21-05637-s001.pdf]

International Journal of
Molecular Sciences

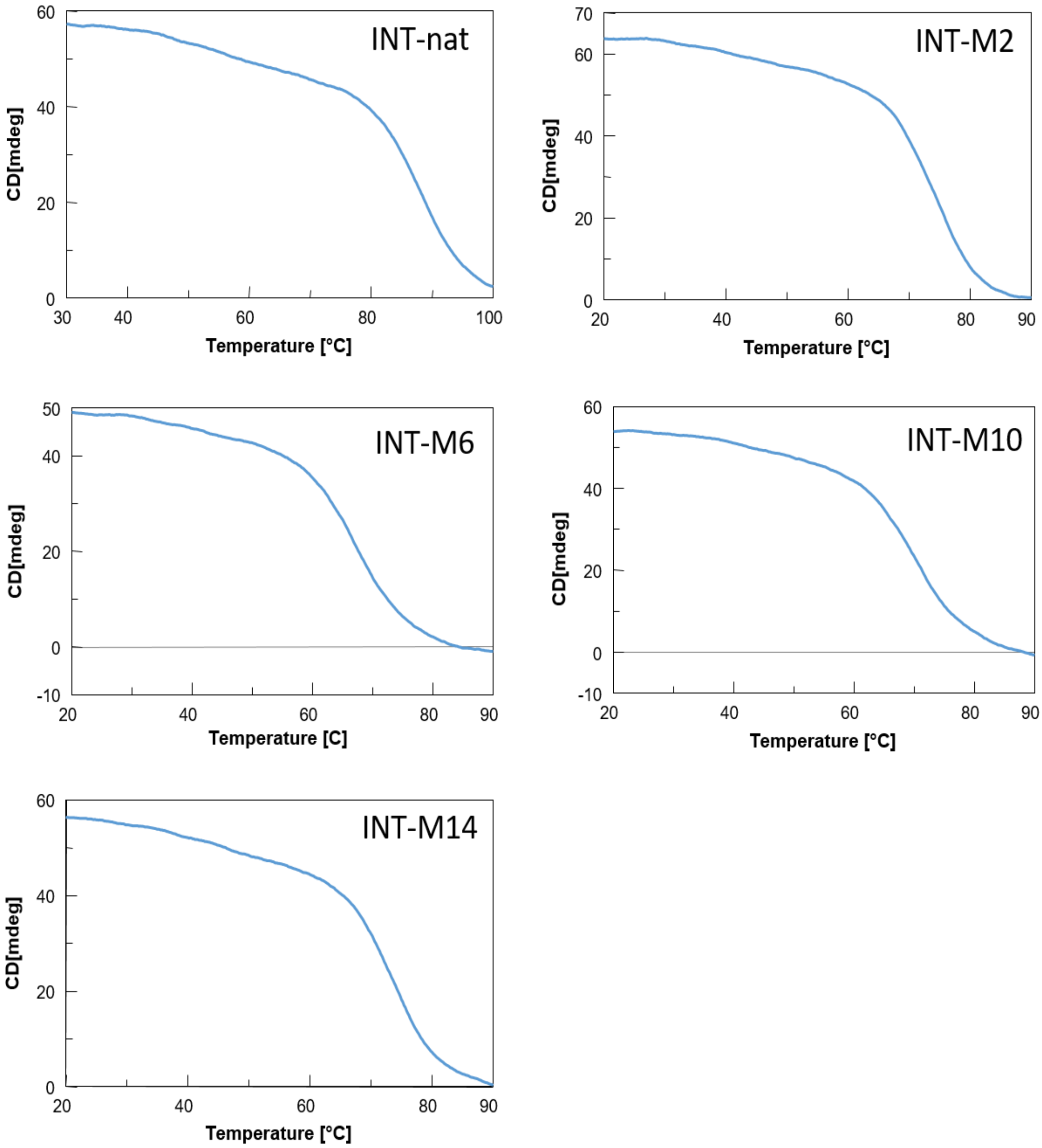

Figure S1. CD melting profiles of the modified ODNs at low potassium ion concentrations (5 mM KCl). See Materials and Methods for experimental details.

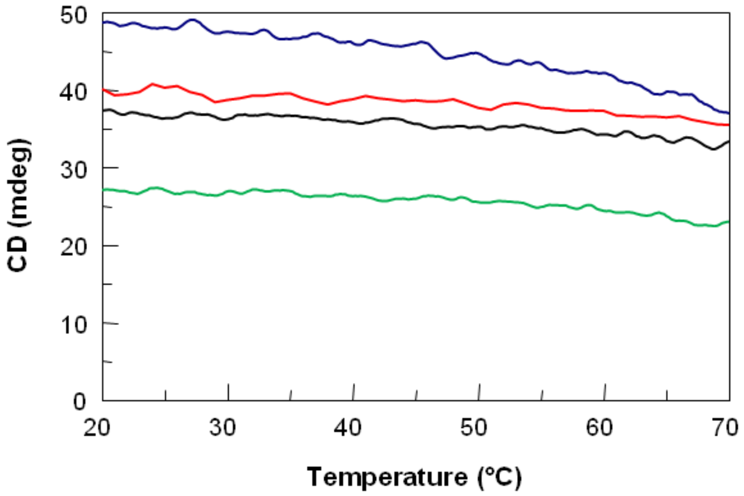

Figure S2. CD heating profiles of INT-M2 (blue), INT-M6 (green), INT-M10 (red) and INT-M14 (black) in a buffer 10 mM HEPES, 40 mM $MgCl_2$, 1% Glycerol, pH 7.5. See Materials and Methods for experimental details.

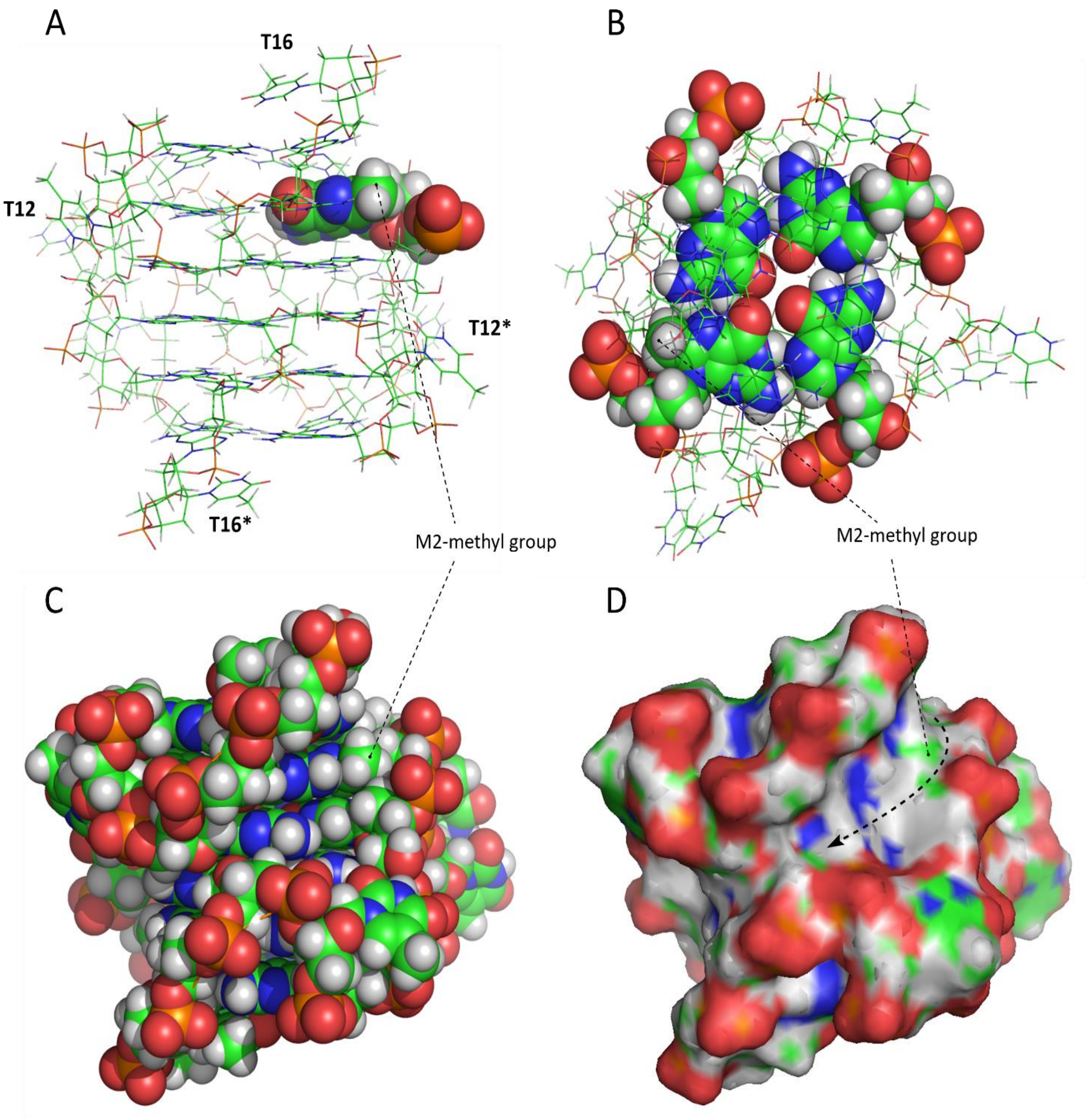

Figure S3. G-quadruplex structure adopted by INT-M2. (**A**) Side view of the stick model. The M-residue is reported in CPK. (**B**) Top view of the stick model. The central G-tetrad is in CPK. (**C**) Side view of the CPK model. (**D**) Side view of the surface model. The arrowed dashed-line indicates the groove in which the methyl group is positioned. Heavy atoms are shown with different colors (carbons, green; nitrogens, blue; oxygens, red; hydrogens, white; phosphorus, orange).

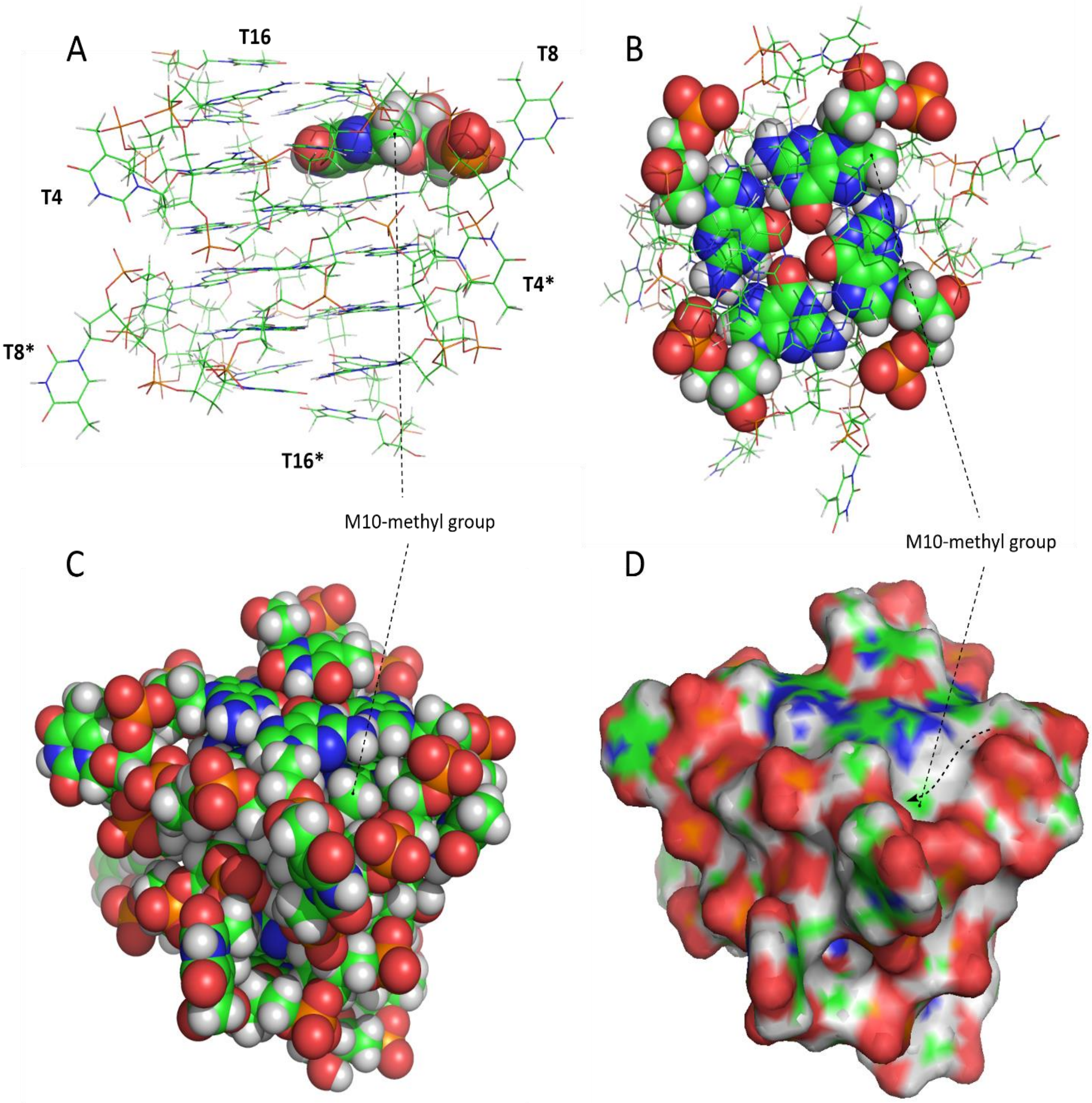

Figure S4. G-quadruplex structure adopted by INT-M10. (**A**) Side view of the stick model. The M-residue is reported in CPK. (**B**) Top view of the stick model. The central G-tetrad is in CPK. (**C**) Side view of the CPK model. (**D**) Side view of the surface model. The arrowed dashed-line indicates the groove in which the methyl group is positioned. Heavy atoms are shown with different colors (carbons, green; nitrogens, blue; oxygens, red; hydrogens, white; phosphorus, orange).

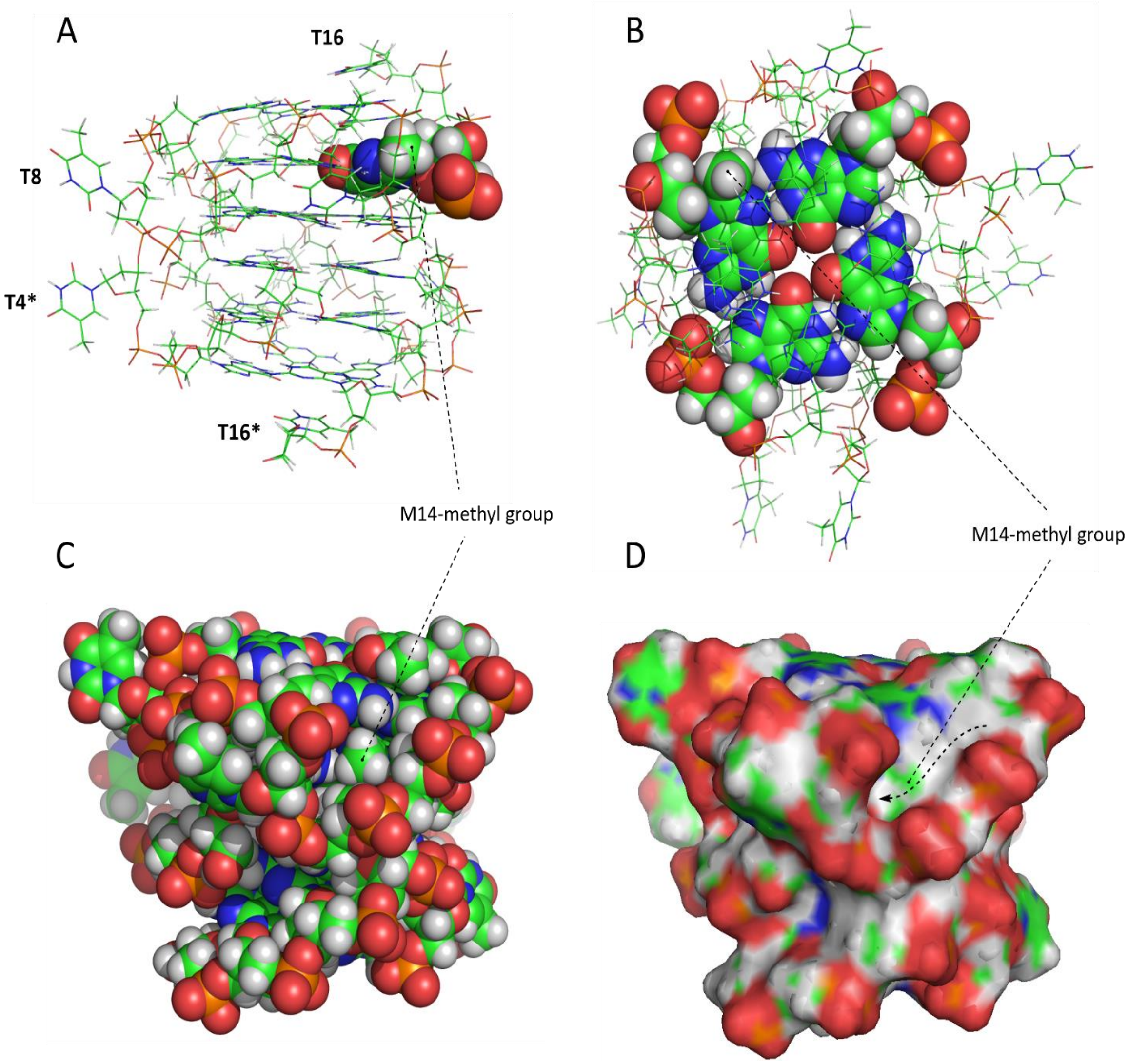

Figure S5. G-quadruplex structure adopted by INT-M14. (**A**) Side view of the stick model. The M-residue is reported in CPK. (**B**) Top view of the stick model. The central G-tetrad is in CPK. (**C**) Side view of the CPK model. (**D**) Side view of the surface model. The arrowed dashed-line indicates the groove in which the methyl group is positioned. Heavy atoms are shown with different colors (carbons, green; nitrogens, blue; oxygens, red; hydrogens, white; phosphorus, orange).
